# Improvement plan for professionalism course guidance based on adult learning theory

**DOI:** 10.15694/mep.2021.000130.1

**Published:** 2021-05-16

**Authors:** Yukihiro Ikeda

**Affiliations:** 1 Kindai University Faculty of Medicine

**Keywords:** Professionalism, first-year student, program improvement

## Abstract

This article was migrated. The article was marked as recommended.

Professionalism is widely known as extremely important to the practice of medicine. However, a methodology has not been established in Japan, such that teachers-in-charge at medical schools are using personal strategies in conducting classes. Evidently, research on professionalism education in Japan is lacking. Therefore, the study aims to report an improvement plan for professionalism education in a certain medical school in Japan with emphasis on practice. Specifically, the study focuses on problem extraction and targets medical students in the first year. The problems of the program were extracted for learner, grouping, facilitator, and presentation. Program improvements were implemented for each of these parts to overcome the problems. The result shows that students gained a better understanding of their roles until graduation. In addition, the number of students who answered that this program was the most memorable in 2019 has tripled compared with that in 2018. Regarding the program improvement, the effect of the program cannot be concluded based on this result alone. In the future, these students should be followed up to investigate any changes. In addition, feedback from each facilitator should be investigated as a future endeavor.

## 1. Introduction

Medical schools worldwide practice professionalism education. A stream of research has investigated the learning and evaluation methods for professionalism ( Al-Eraky, 2015; Foucault *et al.,* 2015; Rougas *et al.,* 2015; Shevell, Thomas and Fuks, 2015; Turner *et al.,* 2015; Mak-van der Vossen *et al.,* 2016; AlMahmoud *et al.,* 2017; Reimer *et al.,* 2019). Professionalism is widely known as extremely important to the practice of medicine. However, considering the ethical and cultural aspects of each country ( Papadakis *et al.,* 2005; Braatvedt *et al.,* 2014; Langenfeld *et al.,* 2014; Welch, Brown and Botash, 2017; Franco *et al.,* 2018) is necessary for the application of professionalism. In this regard, a course named professionalism has been started in Japan in recent years. The Japan Society for Medical Education offers a “medical education model core curriculum” ( The Japan Society of Medical Education, 2016) that describes the outline of lessons in the Faculty of Medicine and “professionalism” in the Japanese context. However, a methodology has not been established, such that teachers-in-charge at medical schools are using personal strategies in conducting classes. Evidently, research on professionalism education in Japan is lacking.

In this regard, the Kindai University Faculty of Medicine has created a curriculum on the basis of the medical education model core curriculum. In first year, professionalism is an expected educational outcome of the university. The following subjects are incorporated into the curriculum: ethics and professionalism, basic skills in medical care, medical safety, team medical care, communication skills, understanding the sociality of medical care. Such subjects are taught together with cultivation and English communication skills to cope with internationalization, autonomous continuous learning ability, and problem-solving ability and connection to medical research, which mainly highlight ethics and professionalism, communication skills, and team medicine. The learning objectives of the course are to understand the cognition and behavior of patients and to realize the concept of providing safe and secure medical services. Through the experience of students, simulated patients, and teachers, the study investigates their aptitude, background, and cognition. The expected outcomes of the abovementioned learning goals are as follows:

1. Recognize one’s cognition and behavior during engagement with patients by learning from the experiences of other medical personnel or patients.
2. Act accordingly by utilizing opportunities to support medical services.
3. Link professionalism with career image.
4. Recognize the importance of medical safety and team medical care.
5. Behave and communicate politely in the clinical setting.
6. Set personal goals to achieve by graduation (competence) and a code of conduct as a medical student.
7. Learn from the experience of other members with respect to enable participation in discussions.
8. Summarize personal experiences and reflect on personal and team performance.

In this report, the authors propose a program improvement plan for the first session of professionalism education. The program is set as the foundation for the duration of medical education (6 years). The program is one of the highlights of an off-campus orientation (1 night and 2 days) for new students and is the first class after admission. It is intended to help students envision their ideals as a doctor. However, the topic is abstract and seems to end as an unfulfilled dream. Therefore, students are tasked to undergo a group work entitled “What will you do after graduation?” to promote their vision about their roles as medical students and enable a firm understanding of their vision.

Therefore, the study aims to report an improvement plan for professionalism education in a certain medical school in Japan with emphasis on practice. Specifically, the study focuses on problem extraction and targets medical students in the first year.

## 2. Subjects, practices, problem extraction, and improvement plan

The learners and strategies are presented as follows:

Learners: 113 first year medical students

Strategies: 110-min session (small group study)

• Read the chapter “Heart for Medical Care” from the book entitled “Respect for Heaven, Love People, Live as a Doctor” by Former President Shiozaki (surgeon). Reflect on and envision your future after graduation. Write down at least 10 items (15 min).
• Share with the group (20 min).
• As a group, carefully select and present 10 items (5 min x 11 groups = 55 min).
• After listening to the presentations, compare group items with other those of other groups. Reflect on the items (20 min).

Students revealed “their expected roles until graduation” at the personal and group levels. However, they only shared goals with other students. Thus, no stimulus was provided for further learning. Establishing a solid foundation is necessary for the professionalism education of first year medical students to set their perspective about future learning in the university. For this reason, professionalism education should be improved. From this point of view, students become oriented with expected educational outcomes at the end of group work. However, doing so may not indicate that learning occurred. With the current method, students engage in group work but do not learn as a group. In addition, students were instructed to reflect on lessons learned from the group work after the session. Currently, the 113 first year students are divided into 11 groups with 10 to 11 participants per group. An average of 5 to 7 members is suitable for small-group learning ( Seo and Miki, 2010). In this case, each group has a large number of students, such that they may be less responsible and less likely to participate. Facilitation by teachers is important for small-group learning. At present, however, no training regarding methodology was provided for teachers. As such, the study considers that the teachers lack understanding in implementing the activity and discussion of the group work. Thus, the question of whether the learners are sufficiently stimulated remains. Presentations were given as part of the group work. Initially, the students were listening with interest but eventually grew tired due to the similarity in presentation.

In addition, when studying at a university, group discussions increase, so students need to be able to hold group discussions firmly. To solve these problems, Table 1 provides the changes in strategy in a similar session conducted on the following year.

**Table 1:** Changes in the Professionalism Orientation Program for first year students

	2018	2019
1 Learner	Write down students expected roles until graduation.	Write down goals until graduation and lacking attributes in the present.
2 Grouping	A total of 11 groups were formed with 10 to 11 students per group.	One group was divided into two subgroups with five to six students per subgroup.
3 Facilitator	Facilitators lack understanding in facilitation the activity and discussion of the group work.	The contents of the implementation, objective of the session, and role of the facilitator were distributed in advance. Questions of unknown points were accepted.
4 Presentation	The 11 groups provide group presentations.	Students provided summarized presentations.

## 3. Results and Discussion

During the off-campus orientation, various programs were implemented in addition to the abovementioned program. Afterward, the students were asked: “What was the most memorable program?” Table 2 shows the results before (2018) and after (2019) the program improvement plan. The result shows that students gained a better understanding of their roles until graduation. In addition, the number of students who answered that this program was the most memorable in 2019 has tripled compared with that in 2018.

**Table 2:** Review of out-of-school orientation for new students

Programs	n (%) for 2018	n (%) for 2019
Interaction with 3rd-year students	43 (38.1)	33 (29.2)
Zazen *	22 (19.5)	- (-)
Story from senior residents	17 (15.0)	22 (19.5)
**Expected roles until graduation**	**15 (13.3)**	**45 (39.8)**
Cherry blossom viewing at night	11 (9.7)	12 (10.6)
Others	5 (4.4)	1 (0.9)
Total	113 (100)	113 (100)

* Zazen is a traditional Japanese meditation method, which was not practiced in 2019.

### 3.1 Learners

The students were enthusiastic about taking the initial class in the medical school. In addition, they were given a background of the profession after reading a section of “Heart for Medical Care” before the group work. Thus, the learners are expected to have achieved a certain degree of learning motivation among the five characteristics of adult learning theory proposed by Knowles ( Knowles, 1999). However, many students are seemingly accustomed to “provided learning” up to high school. Of the five characteristics of adult learning theory, readiness of learning, introduction of learning, self-concept, and past experience are particularly lacking among students. For this reason, stimulation in these aspects should be considered. Furthermore, changing the learning objectives, such as from “writing down” to “writing down and reflecting on the aspects that lack attention in the present” is necessary. Learners should break away from the “given learning” that they are accustomed to. The image of learners required after admission is “learners who can learn more independently, self-initiatively, cooperatively, and strategically” ( Ertmer and Newby 1996; Bhutta *et al.* 2010). The professionalism course should be improved, such that students can change their learning mindset.

Therefore, goal setting should be changed to “write down your expectations of your roles by the sixth year and compare them with the current situation and the lacking aspects.” By slightly improving the goal and expressing it in a concrete manner, the learner can deepen learning. In turn, the session provides the necessary learning opportunity. Next, “peer evaluation” was introduced, where students evaluated their groups. Specifically, the group recognized the students with substantial contribution to the discussion, the positive points of the contribution, and how other members perceived such a remark. Students were evaluated based on three viewpoints. As a result, students became more aware of their contribution to the group work and thus actively participate. In addition, a self-initiated learning attitude ( Braatvedt *et al.,* 2014) by listening to the opinions of members within the group and rebuilding their opinions is expected. In addition, the class is encouraged to reflect and recall their thoughts upon the first reading of the assignment, how their objectives changed through group work, and what they learned throughout. As a result, in addition to giving a bird’s eye view of one’s learning activities, self-reflection is encouraged as well ( Papadakis, *et al.,* 2005). Moreover, as part of the overall review at the end of the session, they reviewed their “their expected roles until graduation” and their evaluation of the entire session experience. By doing so, they are expected to gain growth and maturity and become more proactive and self-reflective. The evaluation of learners is based on whether such perspectives and self-reflections are written appropriately. Positive or negative feedback not only promotes the evaluation of learners’ achievement of learning objectives, but also the monitoring of the program in terms of success and failure, especially the aspects where failure occurred.

### 3.2 Grouping

In 2018, a total of 113 first year students were divided into 11 groups with 10 to 11 members per group. Ideally, the suitable group size for small-group learning is 5 to 7 members ( Seo and Miki, 2010). To meet this requirement, increasing the number of facilitators is necessary. However, doing so is difficult because the number of facilitators is practically limited. A present, the only choice is implementing group work with groups of 10 or 11 students. Therefore, each group was divided into two subgroups and instructed to discuss the same contents. The divided subgroups then hold a discussion as a group. In this manner, the number of members per group remains at 5 to 6, thus increasing proactivity and responsibility for each member.

### 3.3 Facilitator

The facilitator of each group will work closely with the students as a mentor for the next 6 years. In this regard, being a group learning facilitator is considered useful for building trusting relationships with students in the future. To render the group study beneficial, conducting an FD in advance is necessary. However, the period from the mentor’s assignment to this session is short (less than a week). Therefore, the contents of the implementation, objective of the lesson, and role of the facilitator should be distributed in advance, and questions of unknown points will be accepted. In addition, the facilitators should continue to ask students questions during group work as deemed necessary. After the group work, each group facilitator will add a short comment on the good points and positive feedback regarding the discussion. By implementing these improvements, each teacher will become inevitably and actively involved in the group work and observation of the students. Thus, a positive relationship can be established between the teachers and students. In addition, one facilitator provided feedback on the positive and negative aspects of the program after the session. In this regard, time should be allocated for listening to the facilitator’s feedback to further improve the program.

### 3.4 Presentation

As previously mentioned, the monotony of the presentation among the 11 groups is a factor that diminishes the learner’s interest. To this end, improving the format of the presentation is necessary. As mentioned in the section on grouping, each group was divided into two subgroups, where each subgroup discussed the same contents and shared their view. Afterward, the students presented what the group had discovered through the discussion. In doing so, although the presentations are expected to be similar, the time allocated for the presentation was shortened. Therefore, the learners can listen to all presentations.

Finally, regarding the program improvement, the effect of the program cannot be concluded based on this result alone. In the future, these students should be followed up to investigate any changes. In addition, feedback from each facilitator should be investigated as a future endeavor.

## 4. Conclusion

The study presented problems in and improvement plan of the professionalism education program. Examining the effect of the program on medical students over time is necessary.

## Take Home Messages

• Students are required to shift from high school learning styles to college learning styles.
• The professionalism course requires improvement, such that it can be an effective tool for changing the current learning mindset of students.

## Notes On Contributors

Yukihiro Ikeda, PhD, is an associate professor at the Medical Education Center, Kindai University School of Medicine, Japan. He is qualified for the Japan Society for Medical Education certified Medical Education Specialist (Certification number 146). He is responsible for conducting professionalism education for first-year students (new students) in the university. As a communication education expert, he also teaches medical interview classes. He is also involved in training Simulated Patients. ORCiD: https://orcid.org/0000-0002-2768-4145.

## References

[ref1] Al-Eraky M. M. (2015) Twelve Tips for teaching medical professionalism at all levels of medical education. Med Teach. 37(11), pp.1018–1025. 10.3109/0142159X.2015.1020288 25776227

[ref2] AlMahmoud T. Hashim M. J. Elzubeir M. A. and Branicki F. (2017) Ethics teaching in a medical education environment: preferences for diversity of learning and assessment methods. Med Educ Online. 22(1), p.1328257. 10.1080/10872981.2017.1328257 28562234 PMC5508649

[ref3] Bhutta Z. A. Chen L. Cohen J. Crisp N. (2010) Education of health professionals for the 21st century: a global independent Commission. The Lancet. 375(9721), pp.1137–1138. 10.1016/S0140-6736(10)60450-3 20362799

[ref4] Braatvedt C. Poole P. Merry A. Gorman D. (2014) Fitness to practice of medical graduates: one programme’s approach. N Z Med J. 127(1405), pp.70–77. https://assets-global.website-files.com/5e332a62c703f653182faf47/5e332a62c703f672022fd844_content.pdf( Accessed: 13/10/2020). 25399044

[ref5] Ertmer P. A. and Newby T. J. (1996) The expert learner: Strategic, self-regulated, and reflective. Instructional Science. 24, pp.1–24. http://allianceforlearning.co.uk/wp-content/uploads/2017/03/Ertmer-Newby-The-Expert-Learner.pdf( Accessed: 13/10/2020).

[ref6] Foucault A. Dube S. Fernandez N. Gagnon R. (2015) Learning medical professionalism with the online concordance-of-judgment learning tool (CJLT): A pilot study. Med Teach. 37(10), pp.955–960. 10.3109/0142159X.2014.970986 25336258

[ref7] Franco C. Franco R. S. Lopes J. M. C. Severo M. (2018) Clinical communication skills and professionalism education are required from the beginning of medical training - a point of view of family physicians. BMC Med Educ. 18(1), p.43. 10.1186/s12909-018-1141-2 29558914 PMC5859538

[ref8] Knowles M. S. (1999) What is andragogy?in The Modern Practice of Adult Education, From Pedagogy to Andragogy. Cambridge Adult Education, pp.40–57. http://www.umsl.edu/~henschkej/articles/a_The_%20Modern_Practice_of_Adult_Education.pdf( Accessed: 13/10/2020).

[ref9] Langenfeld S. J. Cook G. Sudbeck C. Luers T. (2014) An assessment of unprofessional behavior among surgical residents on Facebook: a warning of the dangers of social media. J Surg Educ. 71(6), pp.e28–e32. 10.1016/j.jsurg.2014.05.013 24981657

[ref10] Mak-van der Vossen M. C. van Mook W. N. A. Kors J. M. van Wieringen W. N. (2016) Distinguishing Three Unprofessional Behavior Profiles of Medical Students Using Latent Class Analysis. Acad Med. 91(9), pp.1276–1283. 10.1097/ACM.0000000000001206 27119326

[ref11] Papadakis M. A. Teherani A. Banach M. A. Knettler T. R. (2005) Disciplinary Action by Medical Boards and Prior Behavior in Medical School. New England Journal of Medicine. 353(25), pp.2673–2682. 10.1056/NEJMsa052596 16371633

[ref12] Reimer D. Russell R. Khallouq B. B. Kauffman C. (2019) Pre-clerkship medical students’ perceptions of medical professionalism. BMC Med Educ. 19(1), p.239. 10.1186/s12909-019-1629-4 31262283 PMC6604300

[ref13] Rougas S. Gentilesco B. Green E. and Flores L. (2015) Twelve tips for addressing medical student and resident physician lapses in professionalism. Med Teach. 37(10), pp.901–907. 10.3109/0142159X.2014.1001730 25665630

[ref14] Seo H. and Miki Y. (2010) Team-based learning.in Igakukyoiku hakusyo. (in Japanese)pp.177–181. Available at: http://jsme.umin.ac.jp/book/pdf/wpmej-2010-177.pdf( Accessed: 13/10/2020).

[ref15] Shevell A. H. Thomas A. and Fuks A. (2015) Teaching professionalism to first year medical students using video clips. Med Teach. 37(10), pp.935–42. 10.3109/0142159X.2014.970620 25313932

[ref16] The Japan Society of Medical Education . (2016) Medical Education Model Core Curriculum. (in Japanese). https://www.mext.go.jp/component/b_menu/shingi/toushin/__icsFiles/afieldfile/2017/06/28/1383961_01.pdf( Accessed: 13/10/2020).

[ref17] Turner D. A. Fleming G. M. Winkler M. Lee K. J. (2015) Professionalism and Communication Education in Pediatric Critical Care Medicine: The Learner Perspective. Acad Pediatr. 15(4), pp.380–385. 10.1016/j.acap.2015.02.011 25937515 PMC4492831

[ref18] Welch T. R. Brown A. C. and Botash A. S. (2017) Using Student Reflective Narratives to Teach Professionalism and Systems-Based Practice. J Pediatr. 187, pp.5–6 e1. 10.1016/j.jpeds.2017.04.049 28526218

