# Supplementary material for: Improvement plan for professionalism course guidance based on adult learning theory
Source: MedEdPublish (2016). 2021 May 16;10:130. [Version 1] doi: 10.15694/mep.2021.000130.1 (PMC10939534; doi:10.15694/mep.2021.000130.1)
Supplement: Peer review history

# Reviewer report: Reviewer response for version 1

**DOI:** 10.21956/mep.18914.r27033

**Published:** 2021-07-14

**Authors:** Richard Hays

**Affiliations:** 1 James Cook University

This review has been migrated. The reviewer awarded 4 stars out of 5 Professionalism in medicine has some strong global similarities but also some differences based on local culture. This article nicely presents a professional development program that reflects Japanese history and culture, an evaluation and then refinements of the program based on that evaluation. This is an appropriate model to follow. The increase in student interest may reflect the obvious investment in developing the program and listening to student views. The transition from secondary to tertiary education can be challenging for students so making them more active participants is sound preparation for their future. The article could be improved by describing how this program fits into a longitudinal professional development stream through the curriculum (will the messages be lost in later years). A response rate for the initial workshop evaluation is not provided. Also, Table 2 is a little confusing - the numbers seem to reflect the proportion of students who found program activities more memorable, a kind of satisfaction scale. All of the activities seem potentially connected to personal and professional developments - if so, how and how could the connection be improved? I am not sure that there should be a competition. Addressing these issues may merit an additional star.

Reviewer Expertise:

NA

No decision status is available

# Reviewer report: Reviewer response for version 1

**DOI:** 10.21956/mep.18914.r27034

**Published:** 2021-05-19

**Authors:** P Ravi Shankar

**Affiliations:** 1 American International Medical University

This review has been migrated. The reviewer awarded 3 stars out of 5 Thank you for the invitation to review this interesting manuscript. As far as I could understand the professionalism orientation during the Introductory session consisted of a two-hour session where they pen down, share, and reflect on their future after graduation. I am not sure the title paper of the paper is appropriate. It gives the impression that a complete course on professionalism is being addressed while only the improvement plan is only for the first session. The off-campus orientation seems interesting. It will be of interest if the author can share why the orientation is being held off-campus and where is the venue. The standard of written English is good. However, in certain areas, I could not fully understand what the author was trying to say. This may be because of the use of expressions and terms which are not commonly used. The improvement plan has been carefully designed and implemented. I am not sure the terms high school learning styles and college learning styles are appropriate. These days there is an emphasis on self-directed learning even during high school.

Reviewer Expertise:

NA

No decision status is available
